# Effects of different doses of granulocyte colony-stimulating factor mobilization therapy on ischemic cardiomyopathy

**DOI:** 10.1038/s41598-018-24020-y

**Published:** 2018-04-12

**Authors:** Rongchong Huang, Haichen Lv, Kang Yao, Lei Ge, Zhishuai Ye, Huaiyu Ding, Yiqi Zhang, Hao Lu, Zheyong Huang, Shuning Zhang, Yunzeng Zou, Junbo Ge

**Affiliations:** 1 Department of Cardiology, The First Affiliated Hospital of Dalian Medical University, 222 Zhongshan Road, Dalian, 116011 China; 2 Department of Cardiology, Shanghai Institute of Cardiovascular Diseases, Zhongshan Hospital, Fudan University, 180 Feng Lin Road, Shanghai, 200032 China; 3 Institutes of Biomedical Science, Fudan University, 138 Dong’an Road, Shanghai, 200032 China

## Abstract

G-CSF mobilization might be beneficial to ICM, but the relationship between effect/safety and the dosage of G-CSF remains unclear. In this study, 24 pigs were used to build ICM models and were randomized into four groups. Four weeks later, different dosages of G-CSF were given daily by subcutaneous injection for 5 days. Another 4 weeks later, all the animals were sacrificed. Electrocardiography, coronary arteriography, left ventriculography, transthoracic echocardiography, cardiac MRI, and SPECT, histopathologic analysis, and immunohistochemistry techniques were used to evaluate left ventricular function and myocardial infarct size. Four weeks after G-CSF treatment, pigs in middle-dose G-CSF group exhibited obvious improvements of left ventricular remodeling and function. Moderate G-CSF mobilization ameliorated the regional contractility of ICM, preserved myocardial viability, and reduced myocardial infarct size. More neovascularization and fewer apoptotic myocardial cells were observed in the ischemic region of the heart in middle-dose group. Expression of vWF, VEGF and MCP-1 were up-regulated, and Akt1 was activated in high- and middle-dose groups. Moreover, CRP, TNF-α and S-100 were elevated after high-dose G-CSF mobilization. Middle-dose G-CSF mobilization therapy is an effective and safe treatment for ICM, and probably acts via a mechanism involving promoting neovascularization, inhibiting cardiac fibrosis and anti-apoptosis.

## Introduction

Chronic ischemic heart disease (IHD) is a principal cause of morbidity and mortality worldwide. Recently, advances in both medical and invasive therapies, such as coronary artery bypass grafting and percutaneous coronary intervention, have improved the outcomes in many cases, but the overall prognosis of IHD patients who are admitted to hospital with heart dysfunction remains poor, with a 5-year survival of ∼50% and a 10-year survival of ∼10%^1,2^.

For patients with ischemic cardiomyopathy (ICM), especially for those with multivessel lesions, severe lesions or diffuse stenosis in coronary arteries, there are no therapies available to restore dead myocardium nor to improve cardiac function. On the other hand, although heart transplantation can be regarded as a radical cure for end-stage ICM, it is not appropriate or practical for widespread clinical application in the short term.

During the past few decades, several studies have suggested that stem cell therapy might contribute to the regeneration of infarcted myocardium and enhance neovascularization of ischemic myocardium^1–12^. After myocardial infarction, bone marrow stem cells can spontaneously migrate to ischemic regions and then differentiate into myocardial cells, vascular endothelial cells, or smooth muscle cells according to the different microenvironments in which they localize, which may play vital roles in the regeneration of myocardium and the improvement of cardiac function^13,14^. However, this self-healing mechanism is too weak to completely repair the traumatized myocardium, since the number of migrated bone marrow stem cells is often too limited. From this point of view, the mobilization of hematopoietic stem cells and endothelial progenitor cells (EPC) from the bone marrow into peripheral blood circulation appears to be a key. Although the effect and safety of this method remain controversial, as a minimally-invasive approach to stem cell transplantation, granulocyte colony-stimulating factor (G-CSF) mobilization has been applied for the treatment of acute myocardial infarction (AMI)^15–22^. In face of several prior experiments and clinical trials^1,23–27^, whether this cytokine therapy can really benefit the patients with chronic IHD, especially those with ICM, as well as its probable biological mechanisms remain more evidence to be explored. Moreover, the relationship between G-CSF dosage and its corresponding effects are still unclear.

The aim of this study was to investigate the potential effect and safety of different dosages of G-CSF-mobilized bone marrow stem cells on myocardial regeneration and cardiac function in an animal model of ICM.

## Results

Twenty-two of the 24 pigs survived after placement of ameroid constrictors. The 2 deaths were due to AMI and infectious pericarditis after surgery. One death occurred in control group, the other was in high-dose group. However, survival rates were equal among the groups (*P* > 0.05). No complications, such as cardiac death, AMI, fatal arrhythmia, infection, or heart failure, occurred during G-CSF mobilization.

### Serum G-CSF concentrations after mobilization

Serum G-CSF concentration was measured by ELISA. G-CSF concentration peaked on day 7 after G-CSF mobilization in each group. Serum levels of G-CSF in the animals after mobilization were much higher than those in controls. Due to the dose-effect relationship, the concentration of G-CSF in each group varied significantly (*P* < 0.05, Supplementary Figure S1). In high-dose group (10 μg/kg/d), the G-CSF peak concentration reached a maximum of 720 pg/mL.

### Blood cell count

When routine blood examinations were conducted, leukocytes counts, especially those of neutrophils and monocytes, were found to peak between the 5^th^ and 7^th^ day after mobilization. This trend was partially dose-dependent, but was non-linear. No significant difference was observed in the number of leukocytes, neutrophils, or monocytes between the middle- and high-dose groups (*P* > 0.05). Moreover, numbers of lymphocytes, hemoglobin, and platelets remained unchanged after mobilization (*P* > 0.05). CD34 and CD133 expression was determined by three-color flow cytometry with CD45/PE gating (Supplementary Figure S2). CD34 expression was highest on day 7 after mobilization. Absolutely, there existed a positive relationship between the dose of G-CSF and the expression of CD34. Meanwhile, CD34^+^/CD133^+^ (EPC markers) were also up-regulated with increasing dose of C-GSF (Supplementary Figure S3). Interestingly, there was no significant difference in expression of epicellular markers between the middle- and high-dose group (*P* > 0.05).

### Stenosis in left circumflex artery (LCX) and its collateral circulation

Four weeks after placement of ameroid constrictors, coronary arteriography (CAG) revealed complete occlusions in four pigs. Distal segments of the LCX in all other pigs reached a stenosis diameter of ≥95% (Table 1). Four weeks after G-CSF mobilization (8 weeks after constrictor placement), we performed CAG again, and found that four of the controls and two of the low-dose group developed complete occlusions, which suggested that coronary stenosis in these two groups was not improved. In contrast, coronary stenosis of the pigs in the middle-dose group appeared to be significantly improved. Distal segments of the LCXs in five of the six pigs released to diameter stenosis of 60%–90%. However, the improvement in the high-dose group was not so obvious as in the middle-dose group.

**Table 1:** G-CSF mobilization and coronary stenosis.

	Control (normal saline, 2 mL/d, *n* = 6)	Low-dose (G-CSF, 2.5 μg/kg/d, *n* = 6)	Middle-dose (G-CSF, 5 μg/kg/d *n* = 6)	High-dose (G-CSF, 10 μg/kg/d, *n* = 6)
**Distal segments of ameroid in LCX**				
*4 weeks after modeling*				
Stenosis, %	97.2 ± 3.2	96.8 ± 5.0	96.6 ± 3.8	95.9 ± 3.7
*8 weeks after modeling*				
Stenosis, %	98.8 ± 1.2	96.3 ± 2.1	87.8 ± 3.6^*#†‡^	92.3 ± 4.5^*#†^
**Proximal segments of ameroid in LCX**				
*Before modeling*				
Diameter of referring segment, mm	2.19 ± 0.06	2.18 ± 0.06	2.22 ± 0.05	2.20 ± 0.04
Diameter of ameroid constrictor, mm	2.20 ± 0.04	2.16 ± 0.08	2.25 ± 0.08	2.18 ± 0.06
*4 weeks after modeling*				
Diameter of referring segment, mm	2.18 ± 0.07	2.18 ± 0.04	2.21 ± 0.05	2.21 ± 0.06
Minimum diameter, mm	1.49 ± 0.18	1.46 ± 0.20	1.47 ± 0.12	1.50 ± 0.14
Stenosis, %	32 ± 5	33 ± 6	34 ± 5	32 ± 7
*8 weeks after modeling*				
Diameter of referring segment, mm	2.19 ± 0.05	2.18 ± 0.07	2.21 ± 0.06	2.20 ± 0.07
Minimum diameter, mm	1.03 ± 0.14^*^	1.17 ± 0.10^*^	1.32 ± 0.12^*^#^†‡^	1.19 ± 0.15^*^
Stenosis, %	52 ± 8^*^	46 ± 6^*^	41 ± 7^*#†‡^	47 ± 6^*^

LCX, left circumflex artery.

*P < 0.05 compared with 4 weeks after modeling; ^#^P < 0.05 compared with control group; ^†^P < 0.05 compared with low dose G-CSF therapy group; ^‡^P < 0.05 compared with high dose G-CSF therapy group.

We also compared the proximal segments of the LCX between each group. Similarly, middle-dose group displayed the mildest stenosis after G-CSF mobilization. While both low-dose and high-dose group showed improving trends without statistical significance.

When analyzing collateral vessels, we found all the pigs were lack of collateral circulation around the obstructive vessel segments 4 weeks post-modeling. After G-CSF mobilization, we observed LCX distal enhancement in each of the pigs from low-dose to high-dose group during CAG.

Except for LCX, the other coronary arteries in each pig had no obvious stenosis during the study.

### Left ventricular function and remodeling

As shown in Table 2, we used left ventriculography (LVG), transthoracic echocardiography (TTE), cardiac magnetic resonance imaging (CMR) and single photon emission computed tomography (SPECT) to evaluate left ventricular function and remodeling in the pigs.

**Table 2:** Left ventricular function and remodeling measured by LVG, TTE, CMRI, and SPECT.

	Control (normal saline, 2 mL/d, *n* = 6)	Low-dose (G-CSF, 2.5 μg/kg/d, *n* = 6)	Middle-dose (G-CSF, 5 μg/kg/d *n* = 6)	High-dose (G-CSF, 10 μg/kg/d, *n* = 6)
**Systolic functional parameters**				
*LVEF measured by LVG*				
Before modeling, %	75.0 ± 1.3	74.7 ± 4.2	73.7 ± 1.2	76.0 ± 3.0
4 weeks after modeling, %	55.8 ± 3.2	54.5 ± 5.4	53.6 ± 2.4	54.7 ± 5.6
8 weeks after modeling, %	51.9 ± 2.1^*^	54.9 ± 1.5^*#^	59.5 ± 2.1^##†‡^	55.5 ± 5.3^*#‡^
*LVEF measured by TTE*				
Before modeling, %	74.6 ± 2.0	74.2 ± 3.5	73.3 ± 2.1	76.0 ± 3.0
4 weeks after modeling, %	53.2 ± 1.6	52.9 ± 3.8	54.7 ± 3.0	53.4 ± 2.4
8 weeks after modeling, %	50.6 ± 3.0^*^	53.2 ± 2.3^*#^	60.4 ± 3.6^##†‡^	55.8 ± 4.7^*#‡^
*LVEF measured by CMRI*				
Before modeling, %	73.2 ± 1.6	72.5 ± 2.3	72.7 ± 3.5	74.0 ± 2.6
4 weeks after modeling, %	52.6 ± 2.7	52.9 ± 3.8	54.7 ± 3.0	53.4 ± 2.4
8 weeks after modeling, %	50.0 ± 3.0^*^	53.1 ± 2.3^*#^	60.1 ± 3.4^##†‡^	53.8 ± 2.5^*#‡^
*LVEF measured by SPECT*				
Before modeling, %	74.8 ± 2.4	74.1 ± 1.6	73.5 ± 2.7	75.6 ± 3.2
4 weeks after modeling, %	54.7 ± 2.2	53.8 ± 4.3	54.0 ± 2.5	54.5 ± 4.8
8 weeks after modeling, %	51.2 ± 2.7^*^	54.3 ± 1.2^*#^	59.3 ± 4.0^##†‡^	55.0 ± 3.4^*#‡^
**Diastolic functional parameters**				
*E/A measured by TTE*				
4 weeks after modeling	1.0 ± 0.2	1.1 ± 0.2	1.0 ± 0.3	1.0 ± 0.2
8 weeks after modeling	0.9 ± 0.2^*^	1.2 ± 0.2^*#^	1.3 ± 0.3^*#^	1.2 ± 0.4^*#^
*Ea/Aa measured by TTE*				
4 weeks after modeling	0.9 ± 0.3	0.9 ± 0.4	0.8 ± 0.4	0.9 ± 0.4
8 weeks after modeling	0.7 ± 0.3^*^	0.9 ± 0.2	1.0 ± 0.2^*#^	0.9 ± 0.3^#^
*E/Ea measured by TTE*				
4 weeks after modeling	12.2 ± 4.2	12.1 ± 4.5	12.5 ± 3.7	12.4 ± 3.6
8 weeks after modeling	12.4 ± 3.4^*^	11.8 ± 2.8^*#^	11.0 ± 3.1^*#†^	11.0 ± 4.5^*#^
**Diameter and volume of left ventricle**				
*LVEDd measured by TTE*				
Before modeling, mm	32.7 ± 3.0	33.0 ± 2.7	34.1 ± 3.7	32.8 ± 2.8
4 weeks after modeling, mm	35.9 ± 2.9	36.3 ± 2.3	36.9 ± 3.2	35.9 ± 1.8
8 weeks after modeling, mm	36.2 ± 1.5	36.0 ± 2.4	35.8 ± 2.0^*^	36.0 ± 2.2
*LVESd measured by TTE*				
Before modeling, mm	21.5 ± 3.0	19.3 ± 1.8	19.6 ± 2.5	20.4 ± 1.7
4 weeks after modeling, mm	23.5 ± 1.9	24.2 ± 2.1	23.8 ± 1.8	22.7 ± 1.5
8 weeks after modeling, mm	25.6 ± 2.6^*^	23.6 ± 2.3^#^	21.0 ±± 1.4^*#†‡^	21.9 ± 1.2^#^
*LVEDV measured by CMRI*				
Before modeling, mm	32.2 ± 2.0	30.6 ± 2.3	31.4 ± 2.2	31.8 ± 2.1
4 weeks after modeling, mm	37.0 ± 3.2	35.7 ± 3.0	36.5 ± 2.5	37.1 ± 2.0
8 weeks after modeling, mm	37.7 ± 2.4	35.9 ± 2.9^#^	35.6 ± 2.0^*‡^	37.3 ± 1.6
*LVESV measured by CMRI*				
Before modeling, mm	8.3 ± 3.7	7.6 ± 2.6	7.9 ± 3.2	8.1 ± 1.6
4 weeks after modeling, mm	13.8 ± 2.6	13.0 ± 1.8	13.2 ± 2.4	14.0 ± 2.7
8 weeks after modeling, mm	15.0 ± 2.0^*^	12.2 ± 2.3^#^	11.6 ± 3.1^*#†‡^	13.5 ± 1.8^#^
**Left ventricular motion**				
*8 weeks after modeling*				
Improved, N	8	7	2^#†‡^	3^#†^
Unimproved, N	2	3	7^#†‡^	3
**Ischemic area**				
*Size of left ventricular infarct measured by CMRI*				
4 weeks after modeling, %	5.2 ± 2.1	4.9 ± 1.6	4.8 ± 2.8	5.1 ± 2.6
8 weeks after modeling, %	6.5 ± 1.5^*^	4.8 ± 2.0^#^	4.0 ± 1.7^*#†‡^	4.7 ± 2.0^#^
*Myocardial perfusion defects measured by SPECT*				
4 weeks after modeling, %	11.2 ± 3.0	10.4 ± 1.4	10.9 ± 2.8	12.0 ± 3.4
8 weeks after modeling, %	15.2 ± 2.5^*^	8.7 ± 2.6^*#^	6.2 ± 2.3^*#†‡^	8.9 ± 2.0^*#^

LVG, left ventriculography; TTE, transthoracic echocardiography; CMRI, cardiac magnetic resonance imaging; SPECT, single photon emission computed tomography; LVEF, left ventricular ejection fractions; E, mitral E peak; A, mitral E peak; LVEDd, left ventricular end diastolic diameter; LVESd, left ventricular end systolic diameter; LVEDV, left ventricular end diastolic volume; LVESV; left ventricular end systolic volume.

*P < 0.05 compared with 4 weeks after modeling; ^#^P < 0.05 compared with control group; ^†^P < 0.05 compared with low dose group; ^‡^P < 0.05 compared with high dose group.

**Figure 1:**
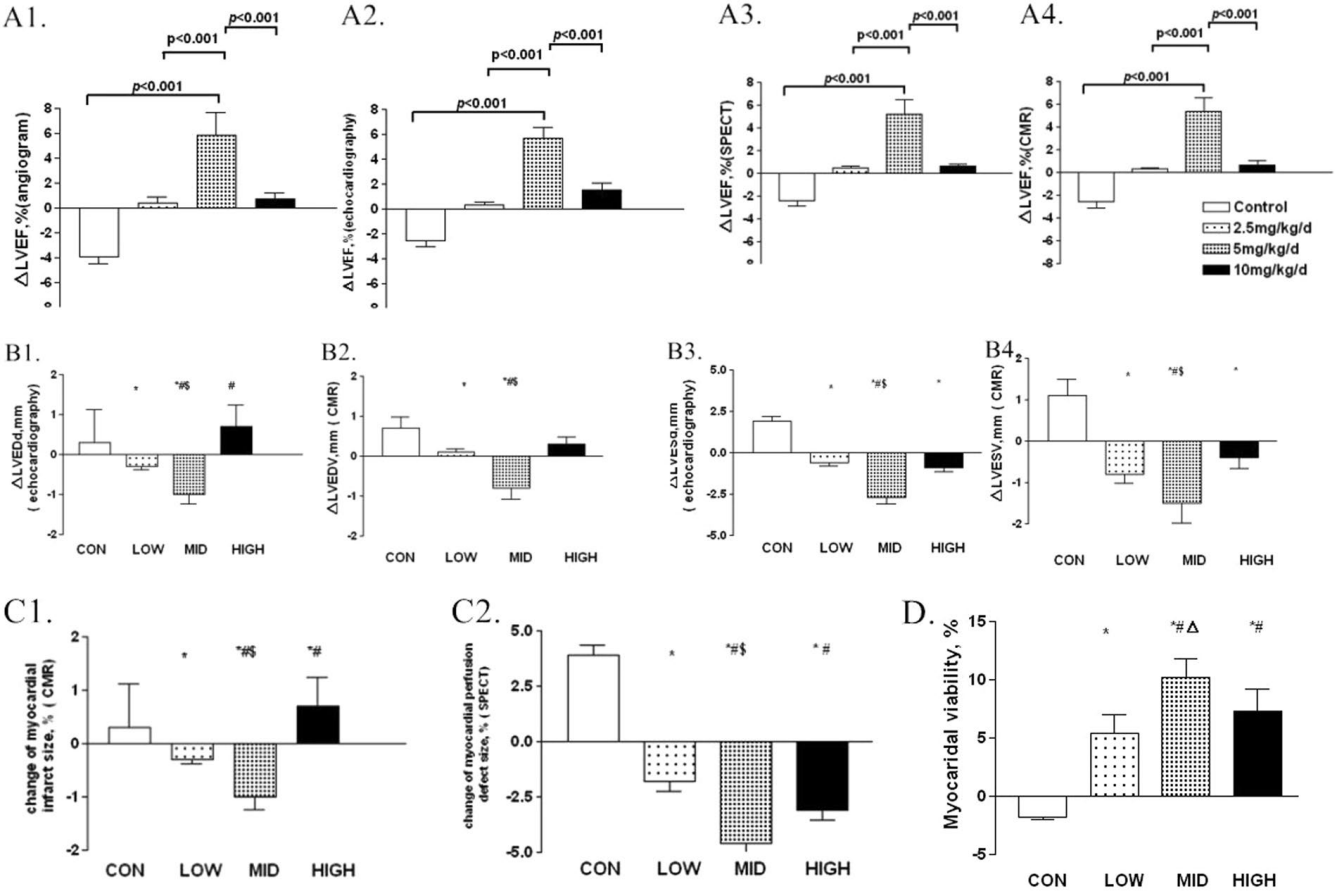
Changes of cardiac function and left ventricular remodeling measured by different means of imaging examinations, after G-CSF mobilization. (**A**) Changes of LVEF measured by different means of imaging examinations, from four to eight weeks post-modeling. (**A1**) Changes of LVEF measured by left ventriculography (LVG). (**A2**) Changes of LVEF measured by transthoracic echocardiography (TTE). (**A3**) Changes of LVEF measured by cardiac magnetic resonance imaging (CMR). (**A4**) Changes of LVEF measured by single photon emission computed tomography (SPECT) ΔLVEF = change of LVEF. (**B**) Changes of left ventricular diameters and volume measured by TTE and CMR, from four to eight weeks post-modeling. (**B1**) Changes of left ventricular end-diastolic diameter measured by TTE. (**B2**) Changes of left ventricular end-diastolic volume measured by CMR. (**B3**) Changes of change of left ventricular end-systolic diameter measured by TTE. (**B4**) Changes of left ventricular end-systolic volume measured by CMR ΔLVEDd = change of left ventricular end-diastolic diameter, ΔLVEDV = change of left ventricular end-diastolic volume, ΔLVESd = change of left ventricular end-systolic diameter, and ΔLVESV = change of left ventricular end-systolic volume. CON = control, LOW = low dose group, MID = middle dose group, and HIGH = high dose group. *p < 0.05 compared with control group, ^#^p < 0.05 compared with low dose group, and ^$^p < 0.05 compared with high dose group. (**C**) Changes of myocardial infarct and perfusion detect size measured by CMR and SPECT, from four to eight weeks post-modeling. (**C1**) Changes of myocardial infarct size by CMR. (**C2**) Changes of myocardial perfusion detect size measured by SPECT CON = control, LOW = low dose group, MID = middle dose group, and HIGH = high dose group. *p < 0.05 compared with control group, ^#^p < 0.05 compared with low dose group, and ^$^p < 0.05 compared with high dose group. (**D**) Myocardial viability measured by SPECT, eight weeks post-modeling. CON = control, LOW = low dose group, MID = middle dose group, and HIGH, high dose group. *p < 0.05 compared with control group, ^#^p < 0.05 compared with low dose group, and ^Δ^p < 0.05 compared with high dose group.

Baseline left ventricular ejection fraction (LVEF) detected by each measurement was balanced among the groups. Since ameroid constrictors were fitted, LVEF dropped equally in each group. Eight weeks after placement, we reexamined the LVEF of every pig and found a significant improvement of approximately 10% in middle-dose group (*P* < 0.001, Fig. 1A1). While, compared with the controls, no difference of LVEF could be detected in either low-dose or high-dose group (*P* > 0.05). Parameters of left ventricular diastolic function, such as mitral E peak and A peak, were also detected by TTE (Table 2). Left ventricular diastolic function of the pigs in each G-CSF mobilization group was better than that in control group (*P* < 0.05). However, the middle-dose group showed the greatest improvement.

The values of left ventricular end-diastolic diameter, left ventricular end-diastolic volume, left ventricular end-systolic diameter and left ventricular end-systolic volume, measured via both TTE and CMR, also changed with ischemic myocardium modeling and G-CSF mobilization (Fig. 1A2, Table 2). Compared to low-dose and high-dose G-CSF mobilization, middle-dose G-CSF provided the most benefit (*P* < 0.05).

We also measured left ventricular aneurysm and its paradoxical motion via TTE and CMR. After G-CSF mobilization, both middle- and high-dose group showed a significant improvement, but this improvement was greater in middle-dose mobilized group (*P* < 0.05). When using CMR and SPECT to precisely evaluate the ischemic regions, we found that all three dosages of G-CSF could prevent ischemia and infarct development, but middle-dose (5 μg/kg/d) mobilization could shrink the size of the infarcted or ischemic myocardium best (Fig. 1C1 and C2, Supplementary Figure S4), as well as significantly enhance myocardial viability (Fig. 1D).

### G-CSF mobilization and myocardial histopathology

**Figure 2:**
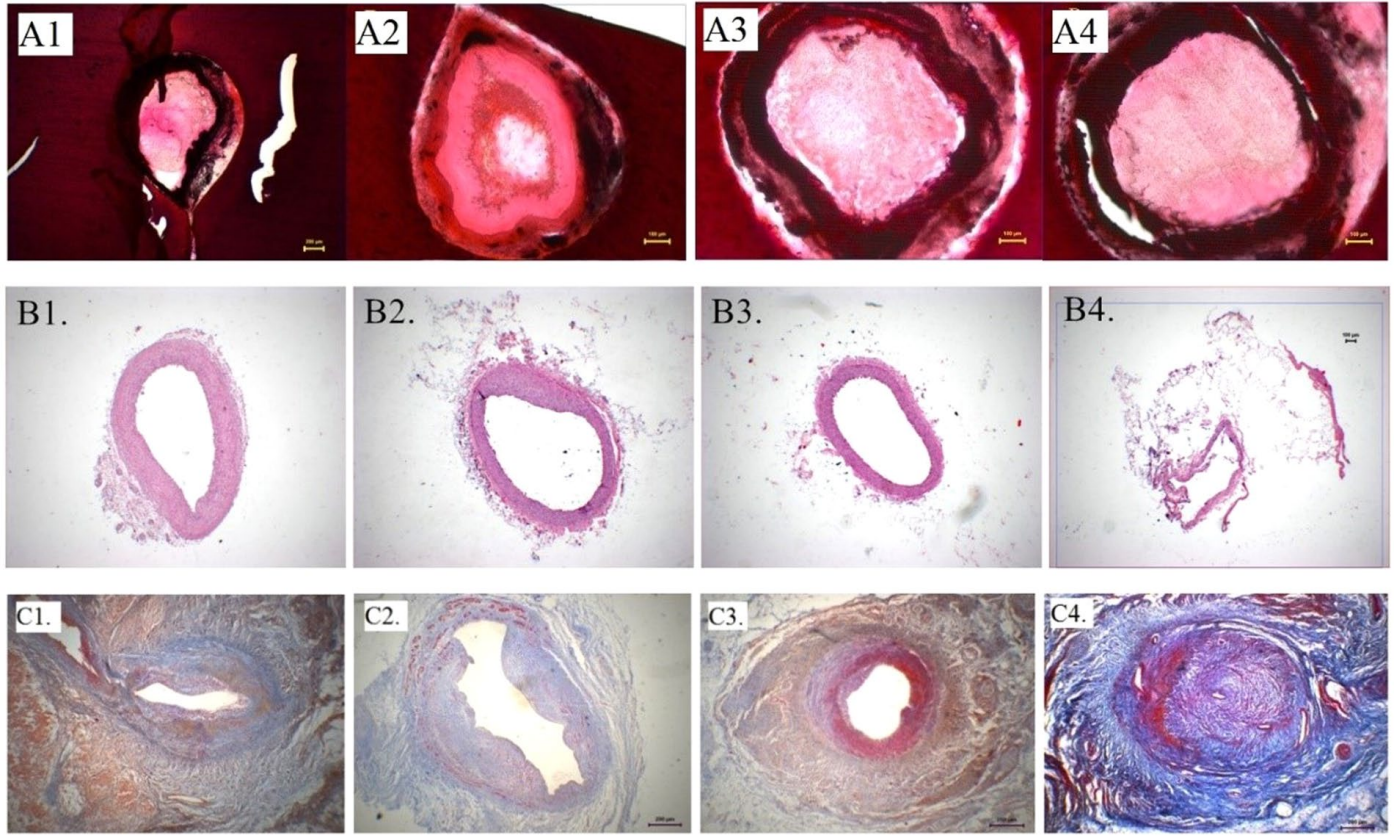
Histopathological examinations, eight weeks post-modeling. (**A**) Ameroid segments in LCX, HE staining, 40×. (**A1**) Control group. (**A2**) Low dose group. (**A3**) Middle dose group. (**A4**) High dose group. (**B**) Proximal segments of ameroid in LCX, HE staining, 40×. (**B1**) Control group. (**B2**) Low dose group. (**B3**) Middle dose group. (**B4**) High dose group. (**C**) Myocardial fibrosis surrounding LCX, Mallory trichrome staining, 40×. (**C1**) Control group. (**C2**) Low dose group. (**C3**) Middle dose group. (**C4**) High dose group.

We performed HE, Mallory and TdT-mediated dUTP nick end labeling (TUNEL) staining to complete the histopathological examination. Pigs in the middle-dose group obviously presented the mildest artery intimal hyperplasia surrounding ameroid segments (Fig. 2A and B), and the least myocardial fibrosis (Fig. 2C).

### G-CSF mobilization and cellular apoptosis

**Figure 3:**
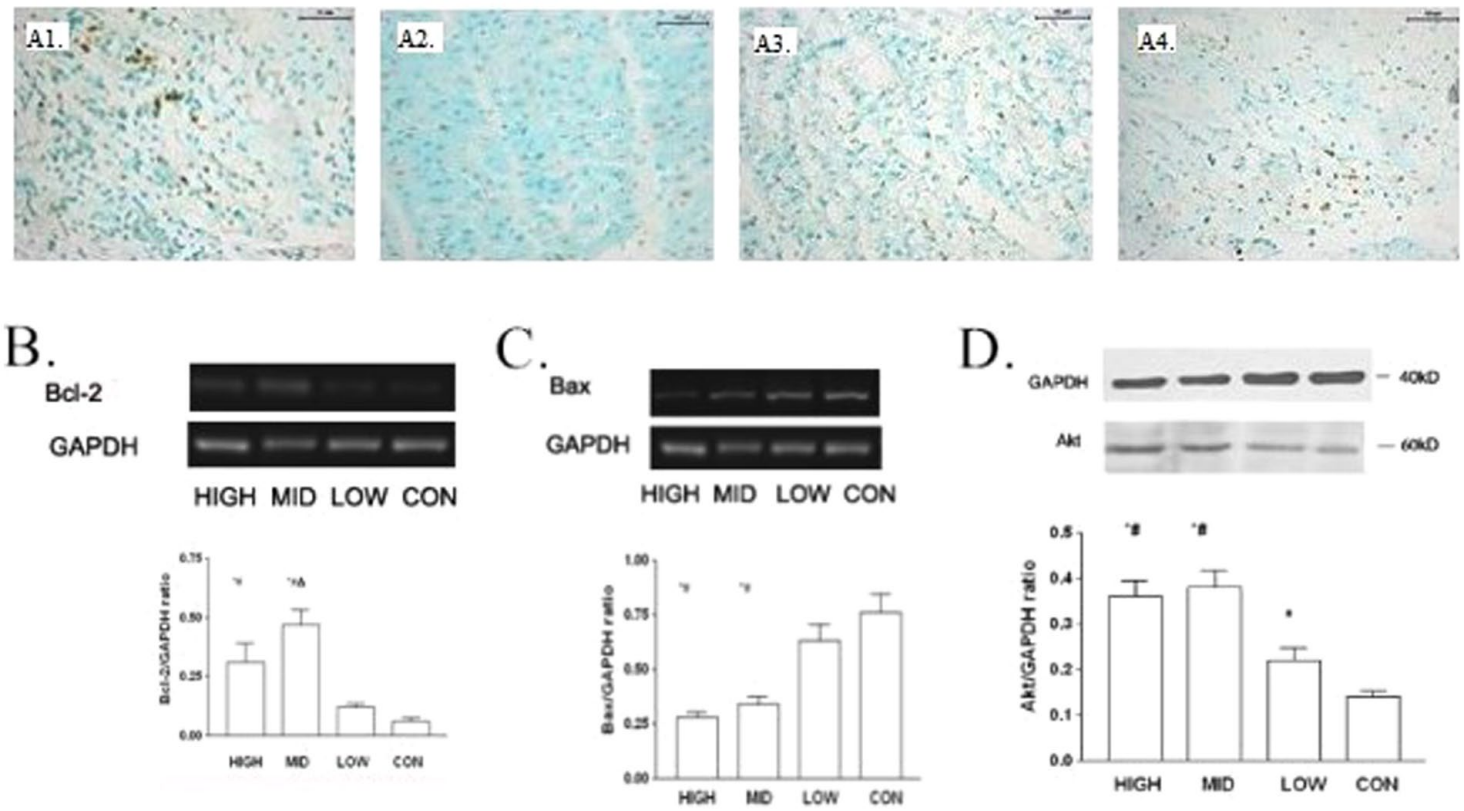
Myocardial apoptosis in infarction border zone, eight weeks post-modeling. (**A**) Myocardial apoptosis in infarct border zone, TUNEL staining, 200×. (**A1**) Control group. (**A2**) Low dose group. (**A3**) Middle dose group. (**A4**) High dose group. (**B**) Expression of Bcl-2 mRNA in infarction border zone measured by RT-PCR CON = control, LOW = low dose group, MID = middle dose group, and HIGH, high dose group. *p < 0.05 compared with control group, ^#^p < 0.05 compared with low dose group, and ^Δ^p < 0.05 compared with high dose group. (**C**) Expression of Bax mRNA in infarction border zone measured by RT-PCR CON = control, LOW = low dose group, MID = middle dose group, and HIGH, high dose group. *p < 0.05 compared with control group, ^#^p < 0.05 compared with low dose group, and ^Δ^p < 0.05 compared with high dose group. (**D**) Expression of Akt in infarction border zone measured by western blot. CON = control, LOW = low dose group, MID = middle dose group, and HIGH, high dose group. *p < 0.05 compared with control group, ^#^p < 0.05 compared with low dose group, and ^Δ^p < 0.05 compared with high dose group.

As shown in Fig. 3A, both middle- and high-dose G-CSF mobilization obviously reduced myocardial apoptosis in the infarct border zone, but the difference between the two groups was not significant statistically. Via western blot and RT-PCR, we observed that Bcl-2 mRNA was up-regulated after middle-dose G-CSF mobilization (Fig. 3B). In contrast, expression of Bax showed an approximately negative correlation with G-CSF dose (Fig. 3C). However, Akt were activated in both high- and middle-dose groups, but in middle-dose group more (Fig. 3D).

### G-CSF mobilization and cell proliferation

**Figure 4:**
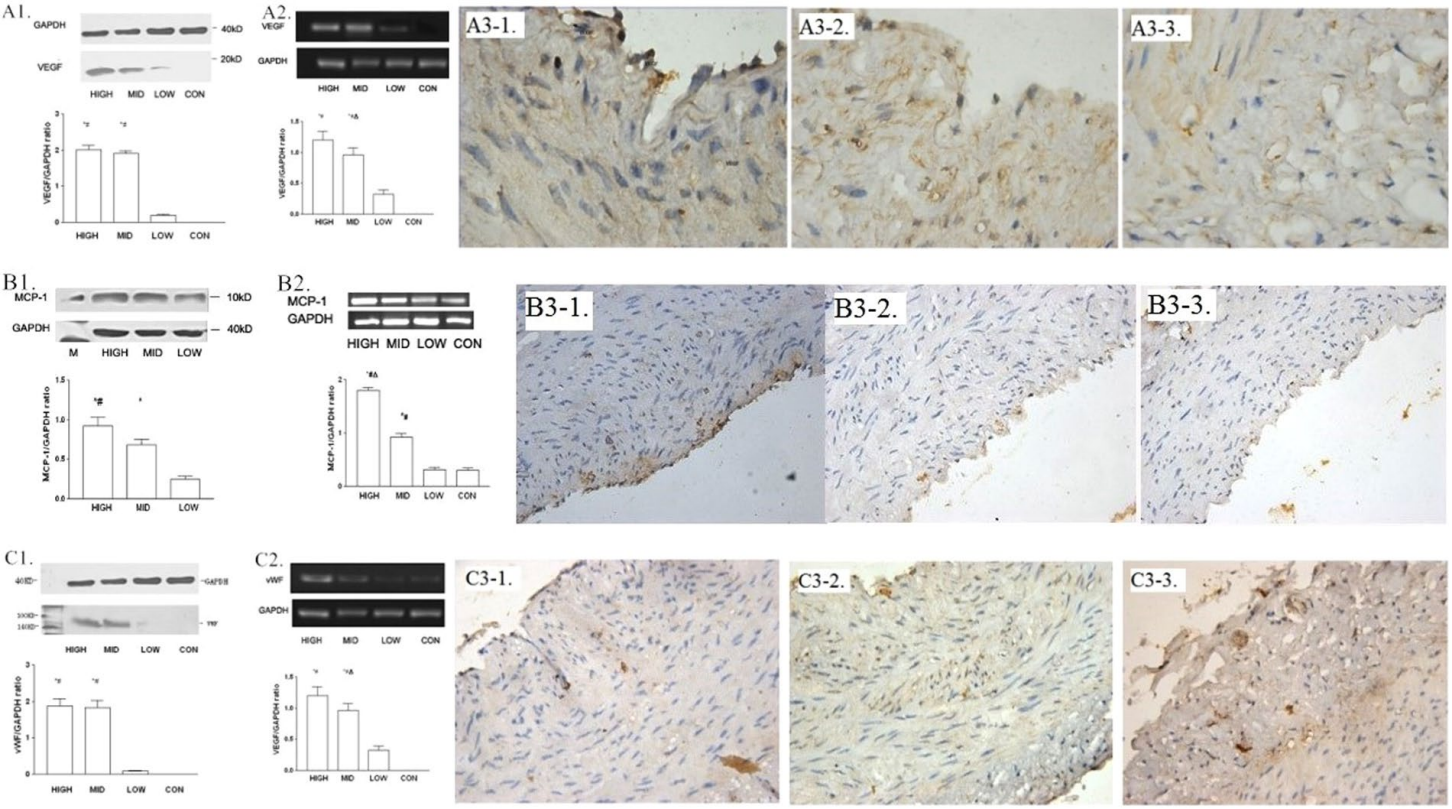
Myocardial proliferation, eight weeks post-modeling. (**A**) Expression of VEGF. (**A1**) Expression of VEGF in infarction border zone measured by western blot CON = control, LOW = low dose group, MID = middle dose group, and HIGH = high dose group. *p < 0.05 compared with control group, and ^#^p < 0.05 compared with low dose group. (**A2**) Expression of VEGF mRNA in infarction border zone measured by RT-PCR CON = control, LOW = low dose group, MID = middle dose group, and HIGH = high dose group. *p < 0.05 compared with control group, ^#^p < 0.05 compared with low dose group, and ^Δ^p < 0.05 compared with high dose group. (**A3**) Expression of VEGF surrounding coronary artery, immumohistochemical staining, 1000×. (A3-1) Low dose group. (A3-2) Middle dose group. (A3-3) High dose group. (**B**) Expression of MCP-1. (**B1**) Expression of MCP-1 in the distal segment of ameriod measured by western blot CON = control, LOW = low dose group, MID = middle dose group, and HIGH = high dose group. *p < 0.05 compared with low dose group, and ^#^p < 0.05 compared with middle dose group. (**B2**) Expression of MCP-1 mRNA in infarction border zone measured by RT-PCR CON = control, LOW = low dose group, MID = middle dose group, and HIGH = high dose group. *p < 0.05 compared with control group, ^#^p < 0.05 compared with low dose group, and ^Δ^p < 0.05 compared with high dose group. (**B3**) Expression of MCP-1 surrounding coronary artery, immumohistochemical staining, 1000×. (B3-1) Low dose group. (B3-2) Middle dose group. (B3-3) High dose group. (**C**) Expression of vWF. (**C1**) Expression of vWF in infarction border zone measured by western blot CON = control, LOW = low dose group, MID = middle dose group, and HIGH = high dose group. *p < 0.05 compared with low dose group, and ^#^p < 0.05 compared with middle dose group. (**C2**) Expression of vWF mRNA in infarction border zone measured by RT-PCR CON = control, LOW = low dose group, MID = middle dose group, and HIGH = high dose group. *p < 0.05 compared with control group, and ^#^p < 0.05 compared with low dose group. (**C3**) Expression of MCP-1 surrounding coronary artery, immumohistochemical staining, 1000×. (C3-1) Low dose group. (C3-2) Middle dose group. (C3-3) High dose group.

**Figure 5:**
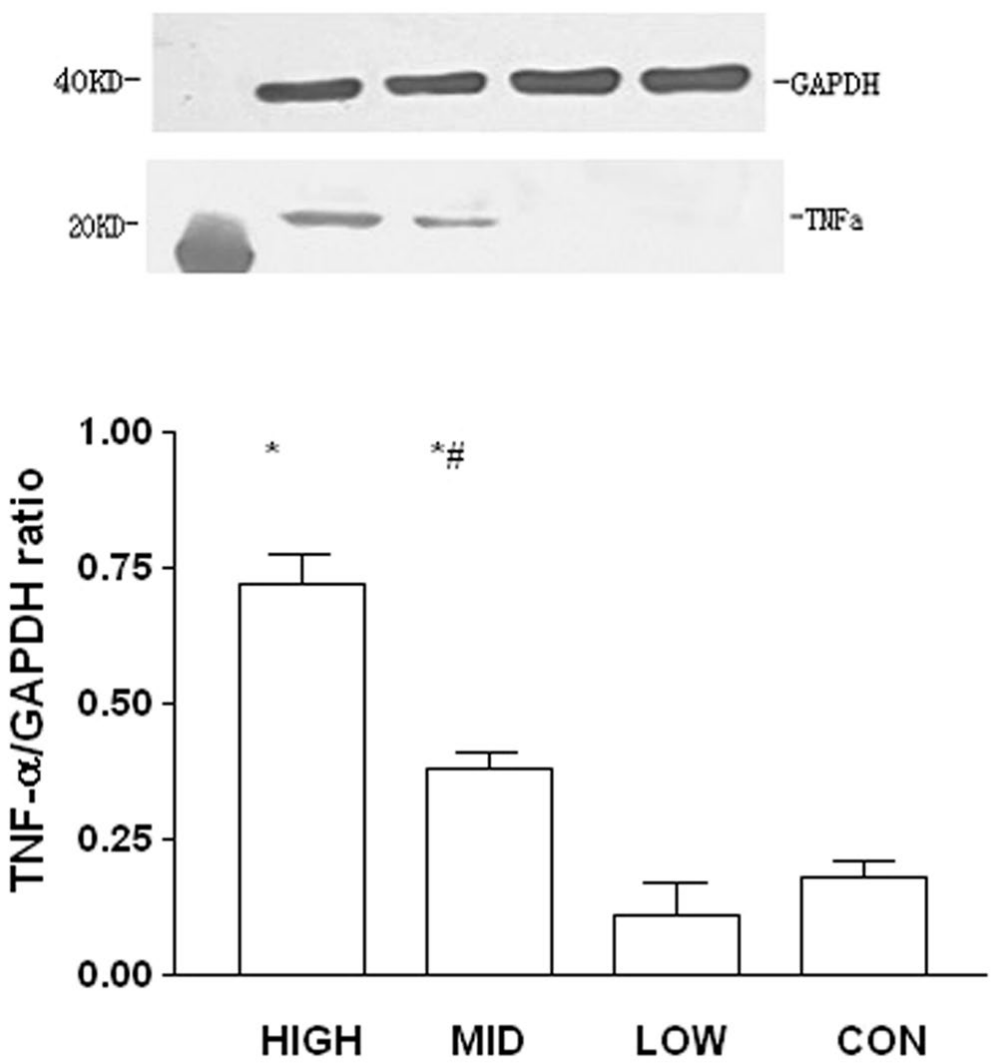
Inflammation in infarction border zone measured by western blot, eight weeks post-modeling. Expression of TNF-α in infarction border zone. CON = control, LOW = low dose group, MID = middle dose group, and HIGH = high dose group. *p < 0.05 compared with control group and low dose group, and ^#^p < 0.05 compared with high dose group.

Western blotting, RT-PCR, and immunohistochemical techniques were used to examine the expression of vascular endothelial growth factor (VEGF) and its mRNA in the infarct zone. As shown in Fig. 4A, VEGF expression remained low in low-dose group, but was up-regulated after both middle- and high-dose G-CSF mobilization. We also evaluated the expression of monocyte chemoattractant protein-1 (MCP-1) and von Willebrand factor (vWF) by the similar means, finding that they were both up-regulated by G-CSF dose-dependently (Fig. 4B–C). However, as an inflammatory marker, tumor necrosis factor-α (TNF-α) was also high-expressed after G-CSF mobilization, especially in high-dose group (Fig. 5). While, we also measured the expressions of peroxisome proliferator-activated receptor-α (PPAR-α) and S-100 in the similar district of the samples, showing that they were correlated to the dose of G-CSF positively (Supplementary Figures S6 and S7).

### G-CSF mobilization and inflammation

Serum concentrations of high-sensitivity C-reactive protein (hs-CRP) and TNF-α were measured by ELISA, to evaluate the relationship between G-CSF mobilization and inflammation. As shown in Table 3, serum concentrations of hs-CRP and TNF-α were elevated, positively correlating with the dose of G-CSF. Meanwhile, we also analyzed the relationship between G-CSF mobilization and other serum biomarkers, such as B-type natriuretic peptide (BNP) and cardiac troponin T (cTNT). The results are shown in Table 3.

**Table 3:** G-CSF mobilization and serum biomarkers.

	Control (normal saline, 2 mL/d, *n* = 6)	Low-dose (G-CSF, 2.5 μg/kg/d, *n* = 6)	Middle-dose (G-CSF, 5 μg/kg/d *n* = 6)	High-dose (G-CSF, 10 μg/kg/d, *n* = 6)
**Hs-CRP**				
Before modeling, g/L	1.20 ± 0.23	1.19 ± 0.20	1.17 ± 0.15	1.23 ± 0.25
4 weeks after modeling, g/L	4.94 ± 0.58^*^	5.04 ± 0.70^*^	4.93 ± 0.47^*^	6.97 ± 0.93^#^
8 weeks after modeling, g/L	8.24 ± 1.25^#†^	10.19 ± 1.46^#†‡§^	15.45 ± 2.16^#†‡^	8.24 ± 1.25^#†^
**TNF-α**				
Before modeling, pg/mL	319 ± 48	328 ± 53	306 ± 61	320 ± 38
4 weeks after modeling, pg/mL	420 ± 52^*^	409 ± 43^*^	437 ± 64^*^	416 ± 43^*^
8 weeks after modeling, pg/mL	538 ± 32^#^	596 ± 65^#^	604 ± 53^#^	598 ± 39^#^
**BNP**				
Before modeling, ng/L	30.0 ± 11.3	32.7 ± 8.3	31.4 ± 10.2	33.8 ± 9.2
4 weeks after modeling, ng/L	68.3 ± 11.3^*^	71.4 ± 12.1^*^	70.5 ± 15.2^*^	92.3 ± 18.6^#^
8 weeks after modeling, ng/L	92.3 ± 18.6^#^	76.8 ± 24.7^†^	57.2 ± 17.5^#†§^	72.0 ± 22.1^†^
**cTNT**				
Before modeling, mg/L	0.06 ± 0.03	0.05 ± 0.02	0.04 ± 0.03	0.05 ± 0.04
4 weeks after modeling, mg/L	0.47 ± 0.11^*^	0.52 ± 0.13^*^	0.48 ± 0.15^*^	0.50 ± 0.16^*^
8 weeks after modeling, mg/L	0.84 ± 0.18^#^	0.34. ± 0.12^#†^	0.20 ± 0.06^#†‡§^	0.31 ± 0.10^#†^

Hs-CRP, high-sensitivity C-reactive protein; BNP, brain natriuretic peptide; cTNT, cardiac troponin T.

^*^P < 0.05 compared with before modeling; ^#^P < 0.05 compared with 4 weeks after modeling; ^†^P < 0.05 compared with control group.

## Discussion

ICM is a common type of IHD, which remains an important cause of cardiovascular death worldwide. During the pathogenesis of chronic disease, decreasing myocardial perfusion leads to ischemia, hypoxia, apoptosis, and fibrosis, gradually resulting in cardiac dysfunction and ventricular remodeling. Since conventional treatments, such as coronary artery bypass grafting and percutaneous coronary intervention, cannot directly salvage tissue from ultimate death, stem cell therapy has been attracting more attention. According to the initial hypothesis of cardiogenesis, ICM is thought to be an attractive potential indication for adult stem cell therapy, which may replace the significant cardiomyocyte loss that occurs^10^.

In recent years, animal experiments and clinical trials have indicated that stem cell therapy may improve cardiac function and prognosis in the patients with acute myocardial infarction^9,19,20,23^, which suggests some practical basis of G-CSF treatment for ICM^16,17^. However, although previous studies have partly explored the effect and safety of G-CSF mobilization regarding ICM, the outcomes remain controversial^28–31^. Several experimental studies have suggested that G-CSF can improve myocardial ischemia and prevent myocardial remodeling via different pathway, which may contribute to the pro-revascularization, anti-apoptosis, and anti-fibrosis mechanisms in ischemic myocardium^32–35^. But evidence from either humans or animals remains limited. To our knowledge, this is the first study to analyze the dose-effect relationship of G-CSF mobilization in an animal model of ICM, and to suggest that appropriate G-CSF mobilization can improve cardiac function and prevent ventricular remodeling.

In current study, after creating ICM pig models by placement of ameroid constrictors, we treated the animals with different dosages (2.5–10 μg/kg/d) of subcutaneous G-CSF daily for 5 days. Four weeks later, we used CAG, LVG, TTE, CMR, and SPECT to compare their outcomes, observing that the pigs in middle-dose (5 μg/kg/d) group had better cardiac function, smaller myocardial infarct size, richer blood perfusion, more surviving myocardium, and milder ventricular remodeling not only than the controls, but also than those treated with other dosages of G-CSF, either higher or lower (2.5 or 10 μg/kg/d). Particularly, no serious complications occurred after G-CSF mobilization during our observation period.

G-CSF can mobilize bone marrow stem cells, including EPCs, to peripheral circulation. These stem cells may play vital roles in the process of ischemia neovascularization^34^. In our study, after administration of G-CSF, the number of CD34^+^/CD45^+^ and CD133^+^/CD45^+^ cells increased in the infarct border zone. At the same time, we found neovascularization increasing but apoptosis of cardiomyocyte decreasing significantly. We inferred that promoting angiogenesis and anti-apoptosis along with anti-fibrosis should be the keys of moderate G-CSF mobilization treating ischemic cardiomyopathy.

In fact, several potential molecular mechanisms have been proposed in some former experiments concerning to acute myocardial infarction, but the exact biologic mechanisms are not fully understood. G-CSF can bind to a cell surface receptor that is a member of cytokine receptor superfamily, which can activate a variety of intracellular signaling cascades such as phosphatidylinositol (PI) 3^−^ kinase, Ras-Raf-mitogen-activated protein (MAP) kinase, Janus kinase (Jak)-signal transducer and activator of transcription (STAT), Src family kinase pathways, and so on^32–38^. Among them, serine/threonine kinase Akt was identified as a downstream target of PI3-kinase. Activation of Akt1 is crucial to the survival of endothelium and myocardium^35,39,40^. When ischemia occurs, the activity of Akt1 can be abolished. In current study, after subcutaneous injection of G-CSF, the reduction of Akt1 expression turned to be weakened, while simultaneously the Bcl-2/Bax pathway is enhanced. Thus, we speculate that G-CSF can drive cellular activities, as well as inhibit myocardial apoptosis.

Moreover, the expression of VEGF, MCP-1, and TNF-α was up-regulated in the same region after G-CSF mobilization. VEGF has been proven to be important in the protection of endothelial stability and function, especially for those atherosclerotic vessels^21,41^. Hence, G-CSF may preserve myocardial viability, rebuild the micro-circulation, and protect endothelial function by regulating these cytokines.

These findings not only support the theories mentioned above, but also provide further practical clues to G-CSF therapy in ICM. We calculate that G-CSF can motivate stem cell homing to ischemic myocardium, ameliorating neovascularization, and recovering ventricular function. Moreover, compared with CD34^+^ cells transplantation, G-CSF mobilization via subcutaneous injection are both more convenient, available and safety during clinical practice, which presents to be a specific advantage.

Interestingly, we found that high-dose G-CSF seemed to have less of a cardiac benefit than middle dose. The levels of inflammatory markers, such as hs-CRP and TNF-α, presented to have a trend of positive correlation to the dosage of G-CSF. As a result, the expressions of these factors were extremely elevated in high-dose group, indicating that high-dose G-CSF mobilization could stimulate an over-activation of inflammation. On the other side, as a marker of dendritic cell (DC), the expression of S-100 was up-regulated with the dosage of G-CSF higher up, showing that high-dose G-CSF mobilization could induce DC filling under endothelial. Prior studies used to confirm DCs and EPCs aggregating under the atherosclerotic plaque^42,43^. While, these two cells are linked with atherosclerosis tightly. Since EPCs, DCs, and mesenchymal stem cells have the same precursor cells with the surface marker of CD34^+^, G-CSF therapy can not only mobilize hematopoietic stem cells and EPCs into peripheral blood, but also increase the number of cells and cytokines associated with inflammation^44^, which can promote cell over-proliferation and cardiac fibrosis^45,46^. Meanwhile, Harada *et al*. previously reported the existence of some G-CSF receptors on the surface of fibroblasts^38^. Once upon ligand binding, the expression of collagen decreases. From this aspect, excessive mobilization of G-CSF may contribute to myocardial fibrosis. In addition, over-expression of VEGF can result in over-proliferation of endothelial and vascular smooth muscle cells, which may contribute to the development of atherosclerosis directly^47,48^. In high-dose group, we have found severe endothelial over-proliferation in the distal segments of ameroid constrictors, which also supports this point of view. Hence, we hypothesize the results of high-dose G-CSF group in our study partly owing to the reasons above.

Another important potential influencing factor of our results is about the expression of TNF-α. Once cardiac infarction, inflammatory response can be activated immediately in the region of ischemic myocardium. As a result, multiple inflammatory factors and adhesion molecules lead to myocardial regeneration and fibrosis^33^. Studies have found that some inflammatory factors, including TNF-α, interleukin-6, and interleukin-1, are associated with ischemic myocardial proliferation^46^, which may also refer to ventricular remodeling, enlargement, and dysfunction. In current study, the expression of TNF-α in infarction border zone have demonstrated to be dose-dependent on the dosage of G-CSF. Thus, we speculate that middle-dose of G-CSF may stimulate TNF-α expressing moderately, which can mobilize bone marrow stem cells homing in ischemic region and can play vital roles in anti-apoptosis and neovascularization. However, high-dose G-CSF may induce TNF-α over-expression, resulting in inflammation and fibrosis excessively.

Although we found some beneficial effects of G-CSF mobilization therapy in pigs with ICM, several issues remain to be addressed. First, since the sample size of our study was limited, a larger-scale animal experiment will be necessary to confirm our findings. Secondly, if possible, the duration of observation should be prolonged. Thirdly, although we confirmed 5 μg/kg/d as the optimal dose of G-CSF to treat the ICM pigs, the exact therapeutic dosage and its safety in real patients requires further investigation. Besides, since we failed to found neonatal cardiomyocyte in the infarct zone while conducting the experiments, mobilized cells seem not to be the primary effectors responsible for the G-CSF effects on the ischemic myocardium^33^. So that, more related signaling pathways need to be detected seriously. Consequently, whether our findings can be directly transferred to clinical practice should be considered cautiously.

Our study has firstly confirmed the dose-dependent effectiveness and safety of G-CSF mobilization therapy in a pig model of ICM. A middle dosage (5 μg/kg/d) of G-CSF mobilization can improve cardiac function, reduce myocardial infarct size, enhance blood perfusion, protect surviving myocardium, and delay ventricular remodeling safely. These findings remind us of balancing G-CSF’s protective effect from angiogenic, anti-fibrosis, and anti-apoptosis and its side-effect such as promoting atherosclerosis and inflammation. Our study may offer new clues to stem cell therapy for the treatment of ischemic heart disease. In future, more details of the biological mechanisms of G-CSF mobilization therapy and clinical application require more evidence and further investigation to clarify.

## Methods

### Animal model of ICM

Twenty-four Yorkshire pigs of either sex weighing 25 to 30 kg (2–3 months old) were anesthetized with intravenous phenobarbital (30 mg/kg) after intramuscular injection with diazepam (1 mg/kg) and ketamine (1.5 mg/kg). Under mechanical ventilation, a left thoracotomy was performed through the fourth intercostal space, using sterile technique. The pericardium was opened, and an ameroid constrictor (Research Instrument Manufacturing, Corvallis, San Diego, USA) of 1.5–2.5 mm internal diameter was placed around the LCX. After observing for a few minutes, pericardium and chest were closed in turn^49,50^. Penicillin (800,000 IU per day) was given intramuscularly for 3 days, and oral aspirin (150 mg per day) was maintained until the animals were sacrificed. The pigs were allowed to recover for 4 weeks after surgery. Electrocardiography (ECG), CAG, LVG, TTE, CMR, and SPECT of dipyridamole 99mTc-MIBI myocardial perfusion imaging (99mTc-MIBI-SPECT) were conducted to evaluate the function of the left ventricle, the myocardial infarct and the perfusion size of every animal. A successful model was defined as one satisfying at least two of the following conditions: (1) newly-presented ST segment depression and (or) T waves changed in leads I, avL and (or) II, III, avF and (or) V4–V6 in ECG; (2) total or sub-total occlusion (a stenosis diameter ≥95%) of the LCX confirmed by CAG; (3) perfusion defects and (or) regional motion abnormalities of the left ventricular lateral walls detected using CMR; (4) perfusion defects and (or) metabolic/perfusion mismatches of the left ventricular lateral walls demonstrated by SPECT. The protocol was approved by the ethics committees of Zhongshan Hospital, Fudan University. All the experiments were performed in accordance with relevant guidelines and regulations.

### Stem cell mobilization

Four weeks after constrictor placement, the surviving successful model pigs were randomly divided into the following four treatment groups: (1) Control group: normal saline, 2 mL/d, subcutaneous injection, 5 days; (2) Low-dose G-CSF therapy group: G-CSF, 2.5 μg/kg/d, subcutaneous injection, 5 days; (3) Middle-dose G-CSF therapy group: G-CSF, 5 μg/kg/d, subcutaneous injection, 5 days; and (4) High-dose G-CSF therapy group: G-CSF, 10 μg/kg/d, subcutaneous injection, 5 days. Another 4 weeks later (8 weeks after constrictor placement), all the animals were sacrificed. with a lethal dose of intravenous potassium chloride.

### Blood and myocardium tissue sampling

Venous blood samples were collected before and after modeling, on day 1, 2, 5 and 7 after the start of G-CSF mobilization, and before sacrifice. From each whole sample, 1 mL was used for analysis of blood cell count by flow cytometry (FCM), then the remainder of each sample was centrifuged (2000 *g*) and the serum was stored at −70 °C until the final analysis. Then ELISA were conducted to measure the serum concentrations of TNF-α, hs-CRP, cTnT, BNP, and G-CSF. While, methods of western blot and reverse transcription-polymerase chain reaction (RT-PCR) were conducted to evaluate the transcriptions and expression of TNF-α, vWF, VEGF, Bcl-2, Bax, MCP-1, and PPAR-α.

After the animals were sacrificed, their hearts were immediately excised. The region of ameroid constrictor placement was visually examined, to confirm circumflex coronary artery occlusion. The myocardium and coronary arteries surrounding ischemic regions were fixed by immersion in 4% formalin and liquid nitrogen, separately.

### Cardiac catheterization and angioplasty

CAG and LVG were conducted before, and at 4 and 8 weeks after constrictor placement via the right femoral artery, left femoral artery and right carotid artery, respectively. After the pigs were anesthetized, a 6 French arterial sheath was inserted for catheterization. CAG was performed in multiple views via 6 French AL1 and JR4 angiographic catheters to confirm coronary stenosis and occlusion, as well as to evaluate the extent of collateral circulation by ‘Rentrop Collateral Class’^51^. LVG was performed via 6 French Judkins pigtail left ventriculography catheter to observe the motion of each ventricular wall.

### Cardiac imaging detection

Before and at 4 and 8 weeks after constrictor placement, cardiac imaging detections were conducted after anaesthesia by the ways mentioned above.

TTE was performed using a GE Vivid 7 (General Electric Vingmed Ultrasound, Horten, Norway), in the left decubitus or supine position. All the measurements were performed according to the recommendations of the American Society of Echocardiography and as described in previous studies^33,50^. Diastolic parameters were measured to coincide with the QRS complex on ECG, while systolic parameters were measured at the point of minimum left ventricle area. The mean of 3 to 5 consecutive beats was used for all measurements in each animal.

All the pigs also underwent CMR and SPECT at similar time-points to the TTE. For CMR examination, pigs were positioned in the body coil of a Siemens MAGNETOM Avanto 1.5 T (Siemens Vision system, Siemens AG, Erlangen, Germany) as described previously^49,52^. ^99m^Tc-MIBI-SPECT imaging was performed using a tri-head SPECT gamma camera (Philips-IRIX, Philips Medical Systems, Milpitas, CA, USA). Pigs were scanned in the supine position with intravenous injection of a mean dose of 370 mBq.

### Histopathologic analysis

After fixation for at least 1 week, tissues from the left ventricles and vessels were sectioned into 6–8 transverse slices. Each slice was weighed, its apical surface was traced and the infarct region was determined visually based on scar tissue. Anterior and posterior wall thickness were determined in multiple transverse sections by measuring endocardial to epicardial distances. Care was taken to determine myocardial wall thickness in the same plane and location in all specimens. Then, samples from each animal were analyzed by hematoxylin–eosin (HE) staining, Mallory trichrome staining and TUNEL staining.

### Statistical analysis

Statistical analyses were performed using SPSS software, version 20.0 (SPSS Inc., IBM Corp., Armonk, NY, USA). Continuous variables are presented as mean ± standard deviation (SD) and were compared using ANOVA, whereas categorical variables are presented as percentages and were compared using the Chi-square test or Fisher’s exact test. All reported *P* values are two-tailed, and a *P* value < 0.05 was considered statistically significant.

## Electronic supplementary material

Supplementary Figures
