# Supplementary material for: Effects of different doses of granulocyte colony-stimulating factor mobilization therapy on ischemic cardiomyopathy
Source: Sci Rep. 2018 Apr 12;8:5922. doi: 10.1038/s41598-018-24020-y (PMC5897440; doi:10.1038/s41598-018-24020-y)
Supplement: Supplementary file 1 — Supplementary Figures [file 41598_2018_24020_MOESM1_ESM.pdf]

Effects of different doses of granulocyte colony-stimulating factor mobilization therapy on ischemic cardiomyopathy

Rongchong Huang, Haichen Lv, Kang Yao, Lei Ge, Zhishuai Ye, Huaiyu Ding, Yiqi Zhang, Hao Lu, Zheyong Huang, Shuning Zhang, Yunzeng Zou, Junbo Ge

Supplementary Figure S1 Serum concentration of G-CSF after mobilization in each group

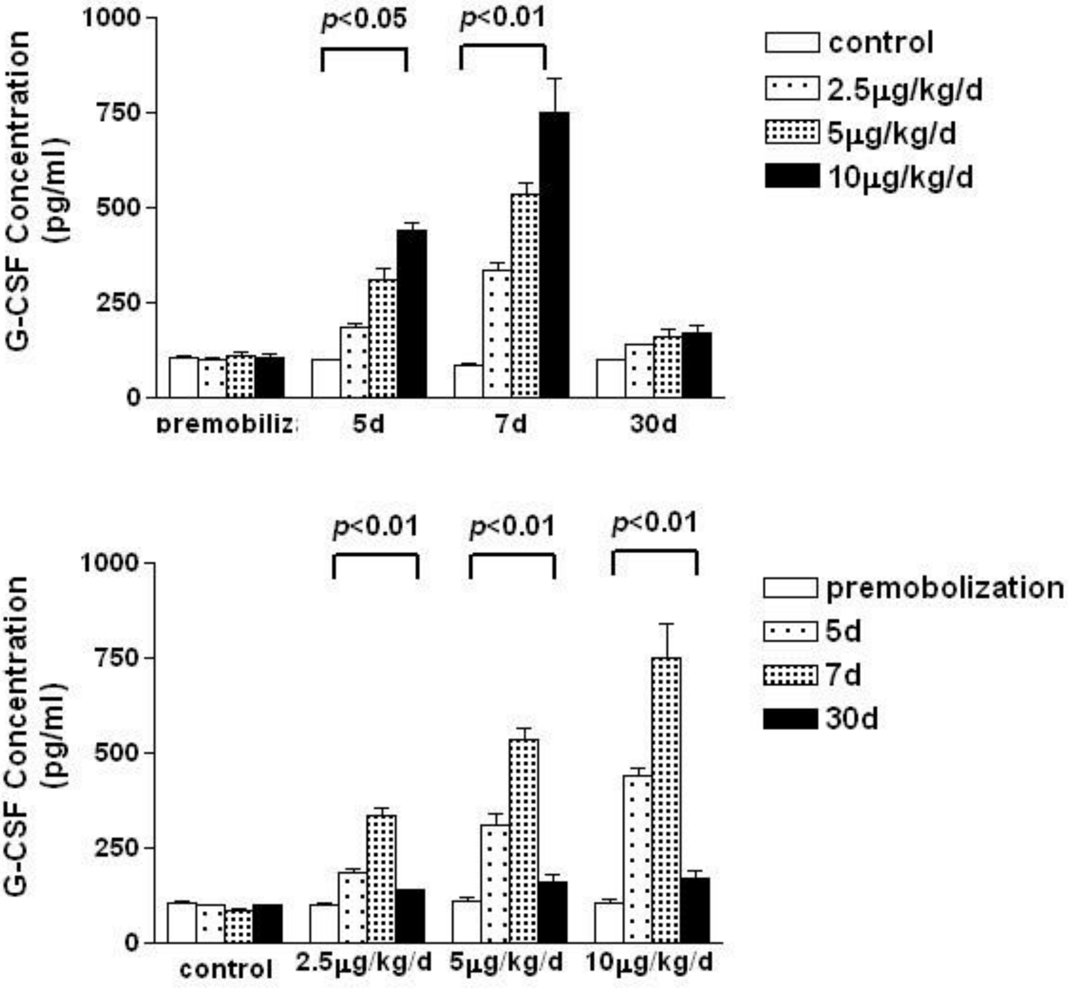

Supplementary Figure S2 Flow cytometry with CD45/PE gating

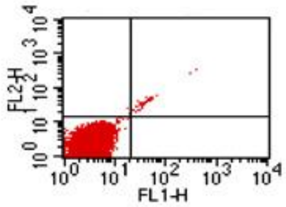

Quad	Events	% Gated	% Total	X Geo Mean
UL	9	0.08	0.08	17.43
UR	102	0.90	0.88	39.22
LL	11221	99.01	96.52	3.85
LR	1	0.01	0.01	21.87

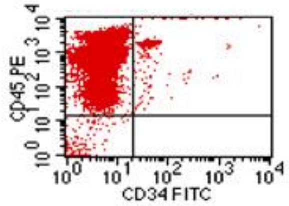

Quad	Events	% Gated	% Total	X Geo Mean
UL	48654	98.33	97.31	5.81
UR	470	0.95	0.94	56.05
LL	355	0.72	0.71	1.80
LR	2	0.00	0.00	24.47

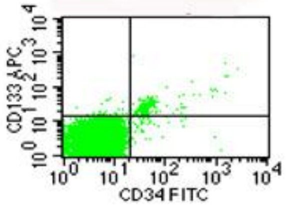

Quad	Events	% Gated	% Total	X Geo Mean
UL	88	0.18	0.18	7.12
UR	384	0.77	0.77	59.37
LL	49097	98.85	98.19	5.75
LR	100	0.20	0.20	45.36

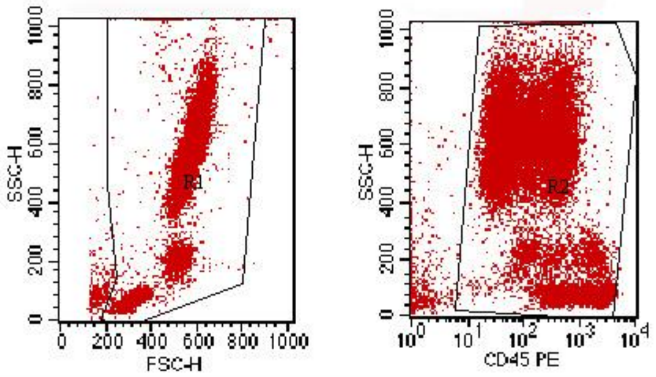

Supplementary Figure S3 Expression of CD34 and CD34/CD133 in each group at the 7th day after G-CSF mobilization

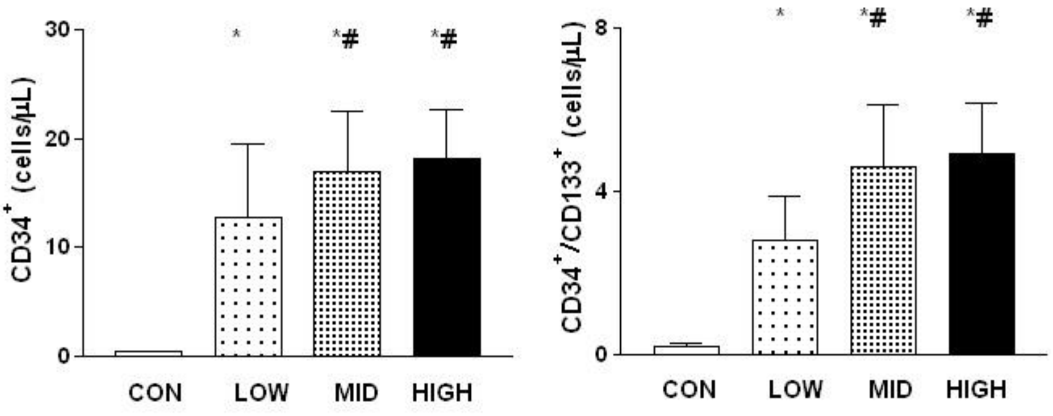

CON, control; LOW, low dose group; MID, middle dose group; HIGH, high dose group; *, $p<0.05$ compared with control group; ＃, $p<0.05$compared with low dose group

Supplementary Figure S4-1 Myocardial perfusion imaging measured by ^{99m}Tc-MIBI-SPECT in moderate dose group

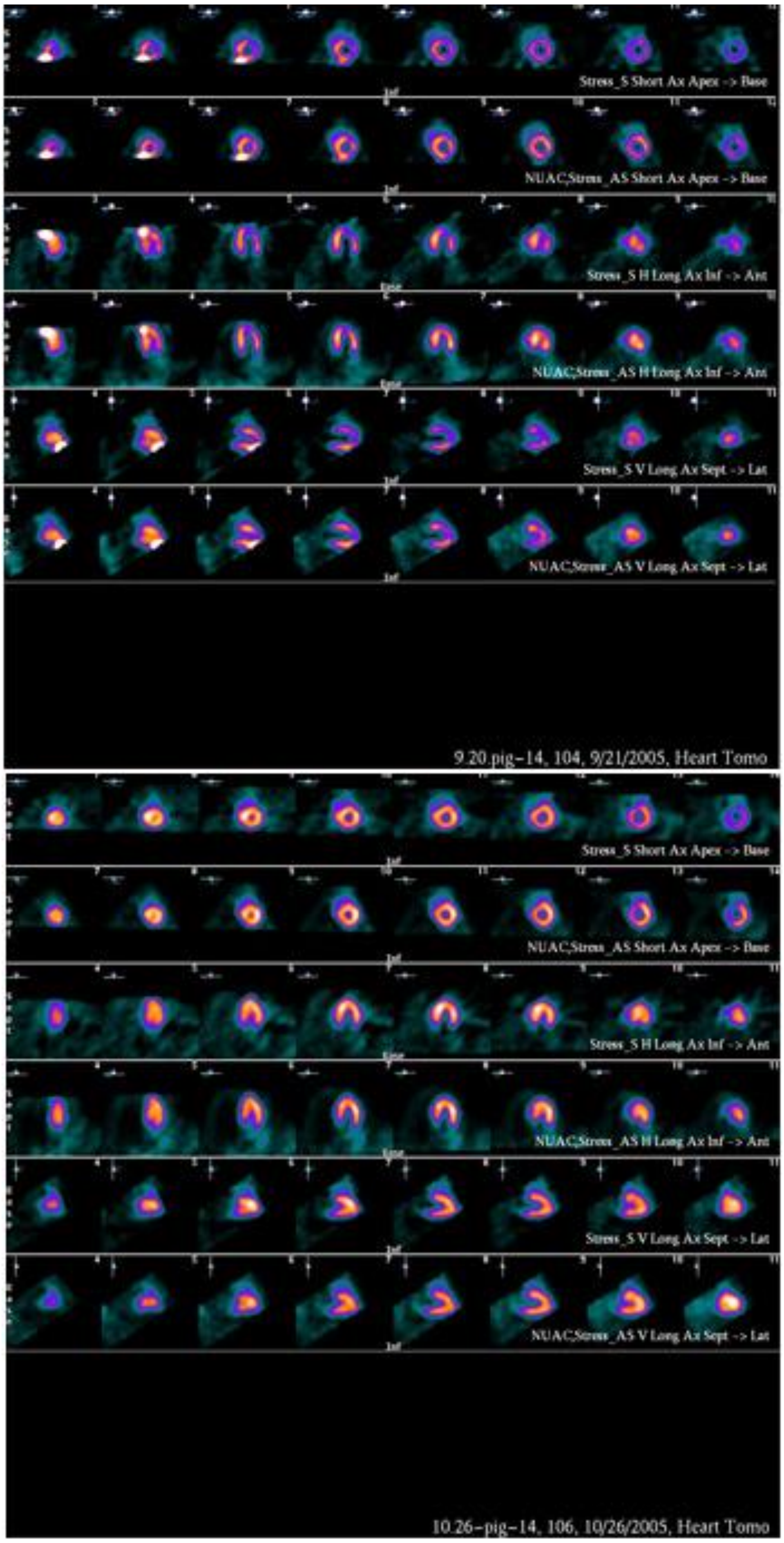

Image above, 4 weeks after modeling; Image below, 8 weeks after modeling.

Supplementary Figure S4-2 Bull's eye plot measured by ^{99m}Tc-MIBI-SPECT in moderate dose group

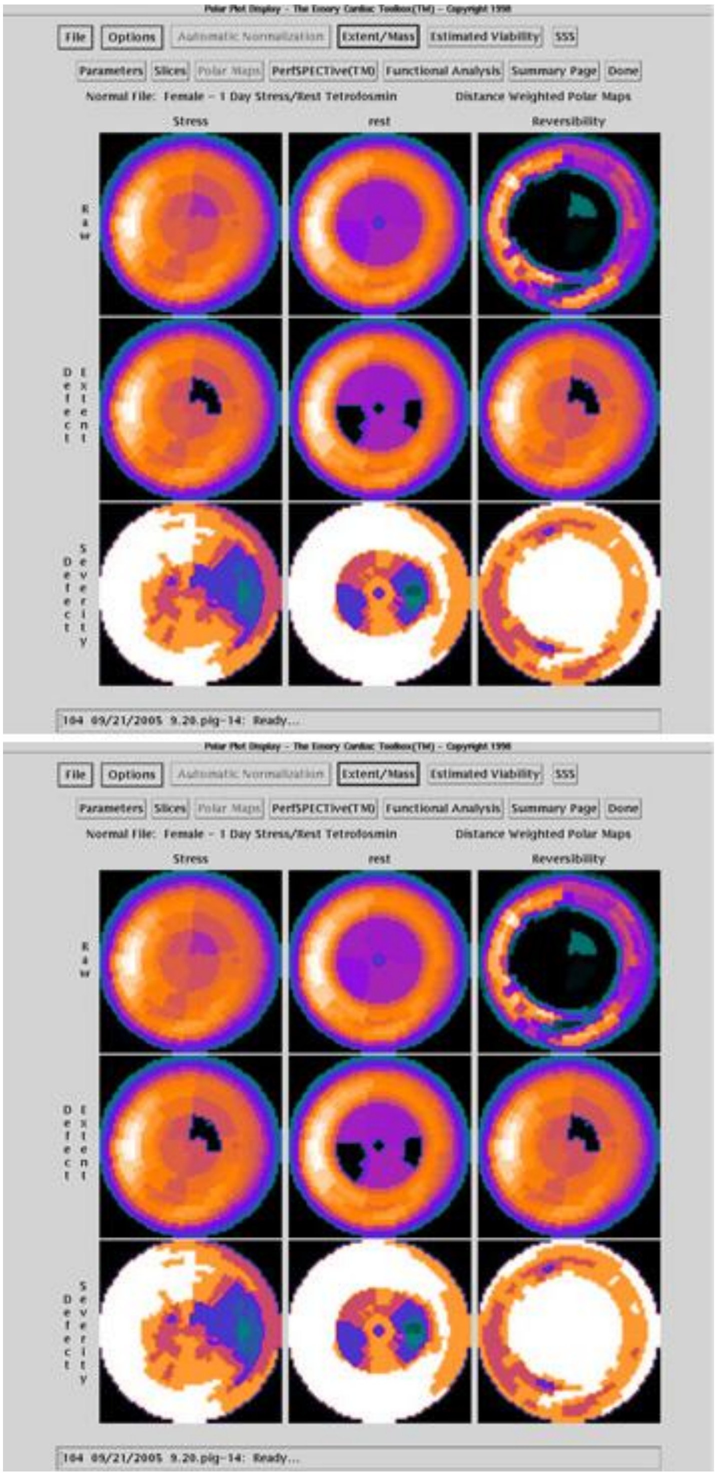

Image above, 4 weeks after modeling; Image, below, 8 weeks after modeling.

Supplementary Figure S5 Expression of Akt in infarction border zone measured by western blot, 8 weeks after modeling

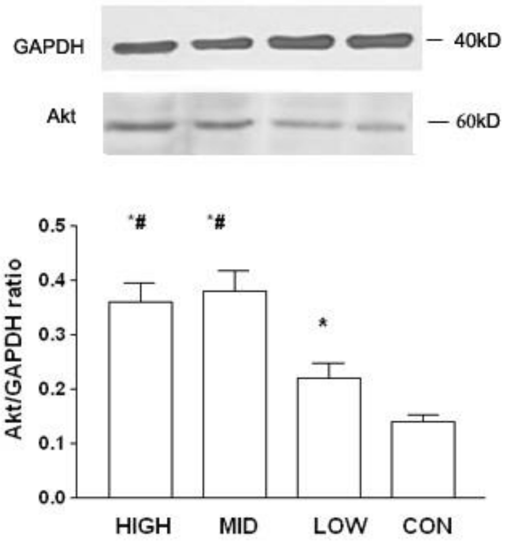

CON, control; LOW, low dose group; MID, middle dose group; HIGH, high dose group.

*, $p<0.05$ compared with control group; ＃, $p<0.05$ compared with low dose group; △, $p<0.05$ compared with high dose group.

Supplementary Figure S6 Expression of S-100 surrounding coronary artery, 8 weeks after modeling (immumohistochemical staining, 200×)

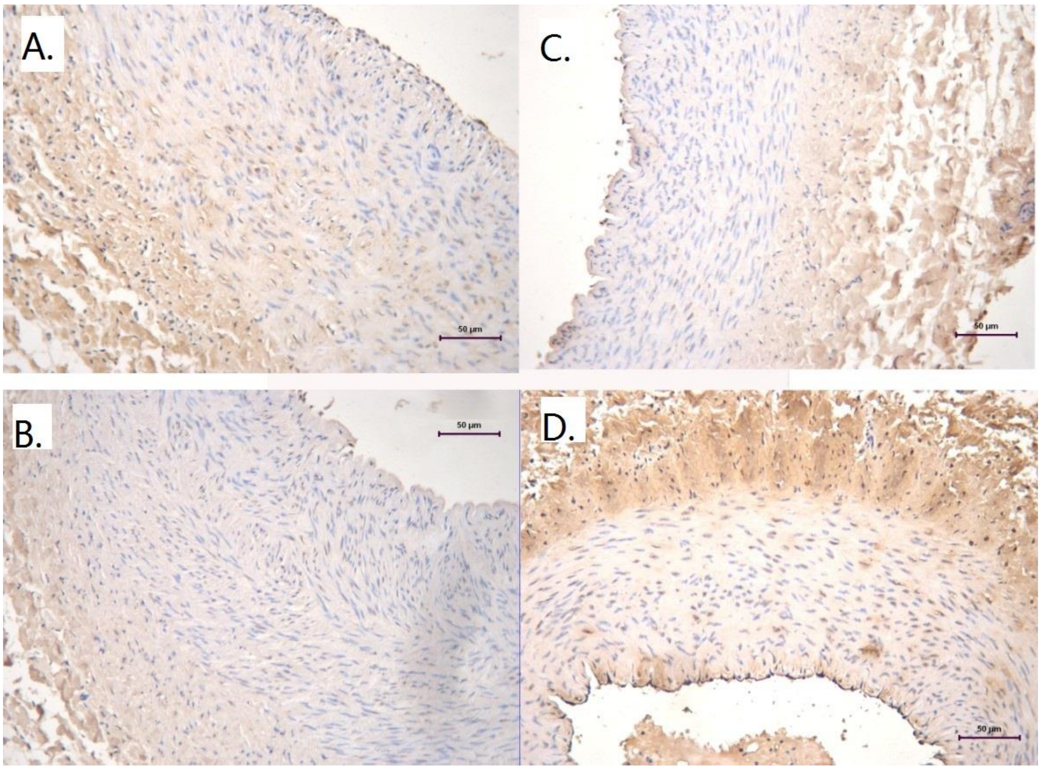

A. Control group, B. Low dose group, C. Middle dose group, D. High dose group.

Supplementary Figure S7 Expression of PPRA-α surrounding coronary artery measured by western blot, 8 weeks after modeling

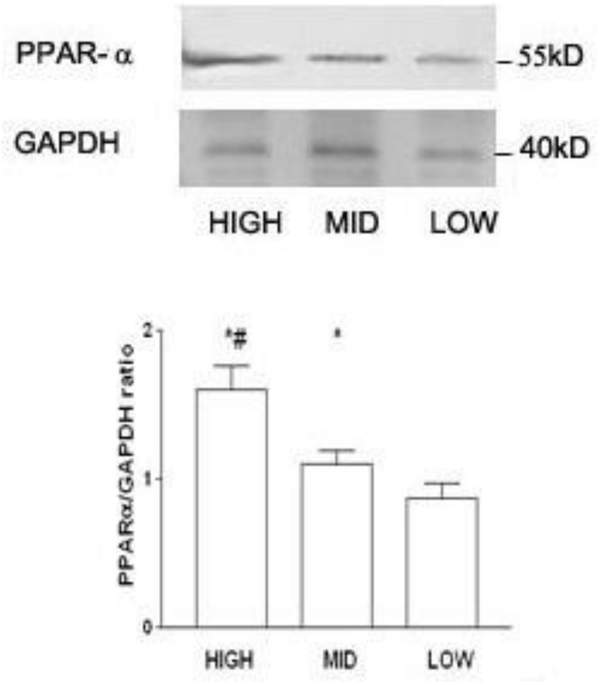

LOW, low dose group; MID, middle dose group; HIGH, high dose group.

*, $p<0.05$ compared with low dose group; #, $p<0.05$ compared with middle dose group.
